# CBCT Evaluation of Alveolar Bone Change and Root Resorption after Orthodontic Treatment: A Retrospective Study

**DOI:** 10.3390/diagnostics14161757

**Published:** 2024-08-13

**Authors:** Silvia Izabella Pop, Diana Cerghizan, Loredana Mițariu, Kinga Mária Jánosi, Antonella D’Andrea

**Affiliations:** 1 Faculty of Dental Medicine, George Emil Palade University of Medicine, Pharmacy, Science, and Technology of Târgu Mureș, 38 Gh. Marinescu Str., 540139 Târgu Mureș, Romania; silvia.pop@umfst.ro (S.I.P.); antonelldandrea6@gmail.com (A.D.); 2 Faculty of Dental Medicine, Lucian Balga University, Bd-ul. Victoriei, 550024 Sibiu, Romania; loredana.mitariu@ulbsibiu.ro

**Keywords:** alveolar bone height, root resorption, periodontium, orthodontic treatment, CBCT

## Abstract

Background: For years, there has been a long debate about the impact of orthodontic treatment on the periodontium of patients. Therefore, it is important to understand the effects of orthodontic forces on the periodontium. The objective of this study was to evaluate the height of the alveolar bone at the four surfaces of specific teeth and the root length of the central incisors before and after orthodontic treatment. Materials and Methods: CBCTs from a group of fifty patients were evaluated before (T0) and after orthodontic treatment (T1). Evaluations of the alveolar bone were performed by measuring the distance from the most apical point of the marginal portion to the CEJ at the buccal (B-ABL), lingual (L-ABL), mesial (M-ABL), and distal (D-ABL) surfaces of the central incisor (CI), first premolar (1st PM), and first molar (1st M). Meanwhile, root resorptions were evaluated by measuring the distance from the center of the tooth at the CEJ to the most apical point of the central incisor. Results: The reduction in the alveolar bone level was highest at the buccal segment (75%) and lowest at the distal (42%) segment, although the decrease was not statistically significant. Root resorption, in terms of reduction in the total length, was detected in the upper central incisor. Conclusions: Fixed orthodontic treatment can produce a significant reduction in root length, but not at the level of the alveolar bone.

## 1. Introduction

Over the course of many years, there has been extensive discussion on the effects of orthodontic treatment (OT) on the periodontium of patients. There are a number of concerns that need to be answered, including whether OT is appropriate for individuals affected with periodontal disease, whether OT alleviates or exacerbates the condition, and what kind of impact it has on the alveolar bone. Hence, it is crucial to comprehend the impact of orthodontic forces acting on the alveolar bone in normal conditions. Generally, OT results in clinically acceptable bone resorption [1]; however, different risk factors can influence this remodeling, negatively determining rarely irreversible periodontal tissue damage, massive bone loss, or even tooth loss [1]. According to Alasqah et al. [2], gender differences, age, and duration of OT treatment are considered risk factors that can influence orthodontic outcomes and modifications of the alveolar bone in various ways. One of the risk factors that orthodontists can control is tooth movement [3]. Orthodontic tooth movement involves a complex sequence of biological tissue remodeling at the level of the periodontal ligament (PDL), which connects the roots to the alveolar bone [4,5,6]. The forces exerted on the teeth generate compression and traction areas at the PDL on one side of the tooth, activating osteoclast activity and giving rise to the resorption of the alveolar bone [4,5,6,7]. At the opposite side of the tooth PDL, areas of tension and stretching will promote the activation of osteoblast-producing bone apposition, contributing to tooth movement [5,8]. A healthy periodontium is crucial to prevent any damage to tissues supporting the teeth during this procedure [8,9,10,11,12]. When there is poor periodontal attachment, changes in the crown–root ratio appear, with fulcrum movement of the tooth being displaced apically, intensification of forces, and consequent prolonged inflammatory processes at the site resulting in root resorption [13]. Furthermore, teeth with smaller and thinner roots feature a thin PDL, leading to the concentration of forces on smaller surfaces and, in the case of more extensive movements, a higher risk of root resorption [5,14]. Therefore, the success of the treatment is dependent on the reaction of the periodontium, and the orthodontist must comprehensively understand its physical characteristics, histology, and anatomy [7,8]. Thus, to achieve optimal results, proper orthodontic tooth movement should be executed with minimal possible iatrogenic effects on the periodontium.

The effects of orthodontic tooth movements on the alveolar bone level and the root length are important aspects to discuss. The duration of treatment is directly proportional to the extent of loss of the marginal part of the alveolar bone; therefore, in addition to root resorption, it will most likely result in a reduction in the alveolar bone height [15,16,17,18,19]. Root resorption can occur, particularly following the continuous application of high forces or when extensive tooth movements are carried out over a more extended period. Yassir et al. have recommended caution be taken in the case of patients with an increased risk of inflammatory root resorption generated by the OT [20].

The teeth commonly considered to be most susceptible to root resorption are the maxillary central incisors (CIs) [13,18]. Several authors have reported a higher incidence of root resorption in the case of OT with tooth extractions [21,22], and others associated it with bracket placement and fixed OT [23,24].

Examinations via intraoral periapical, horizontal and vertical bitewing, and panoramic radiography are used for the assessment of mesial and distal interproximal alveolar bone levels [8,18]. However, these methods have certain disadvantages. Furthermore, while 3D imaging—especially CBCT—improves the detection of buccal and lingual alveolar bone abnormalities, two-dimensional radiography (such as periapical radiographs) has the disadvantage of presenting only the mesial and distal limits of the tooth in a specific axis. Hence, CBCT is not only recommended for pre-existing periodontal diseases and thin alveolar biotypes but, generally, is also suggested for checking the alveolar boundary conditions before and after OT [9,11,19,25,26,27]. On the basis of a large number of articles [10,11,12,17,18,19,20,21,22,23,24,25,26,27,28,29,30,31,32,33,34] on the evaluation of alveolar bone after orthodontic treatment in patients with periodontal disease, major and careful attention should be given to the evaluation of alveolar bone before and after OT in healthy patients. Some studies [10,11,12,30,31,32,33,34] have focused on the evaluation of the marginal alveolar bone by measuring the distance from the cementoenamel junction to the marginal alveolar. In their study, Castro et al. [12] evaluated the buccal and lingual distances between the cementoenamel junction and the alveolar bone crest on CBCT images of the teeth of 30 patients before and after orthodontic treatment.

Son et al. [35] evaluated the changes in palatal alveolar bone thickness for the upper incisors (after retraction and intrusion) in a group of skeletal Class II malocclusion patients who underwent extraction for orthodontic treatment.

With the purpose of increasing knowledge on this topic, CBCT evaluations were conducted, which are considered a useful tool for precise tridimensional measurements, especially when concerns of the patients arise regarding the negative effect of fixed orthodontic therapy [36,37].

The primary objective of this study was to assess, by means of 3D investigation tools (CBCTs), the vertical dimension of the alveolar bone at different sites (buccal, lingual, mesial, and distal) around the central incisors, first premolars, and first molars, along with the root length of central incisors, before and after orthodontic treatment.

## 2. Materials and Methods

This study was conducted in accordance with the Declaration of Helsinki and approved by the Scientific Research Ethics Committee of the George Emil Palade University of Medicine, Pharmacy, Science, and Technology in Târgu Mureș (approval no. 3005). All patients provided written informed consent for participation. The patients’ CBCTs were performed within the Orthodontic Discipline of the Faculty of Dental Medicine, UMFST G.E. Palade Târgu Mureș, and the Natural Smile Clinic, according to the research protocol. Studying alveolar bone levels and root resorption through CBCT investigations has the advantage of 3D analysis of anatomical structures at a high resolution and in several planes, thus contributing to a complete evaluation of each case. However, CBCT investigations must comply with the ALARA principles, and the indication for the use of these investigations must be justified. All measurements were exclusively performed on 3D radiographic images and followed relevant guidelines and regulation protocols.

### 2.1. Study Design, Patient Selection, and Measurements

The sample size for this study was determined using G*Power version 3.1.9.6. software (Franz Faul, Universität Kiel, Kiel, Germany). The calculations indicated that a minimum of 42 samples would be necessary; this size would provide greater than 95% power to detect significant differences, with an effect size of 0.80 at a significance level of α = 0.05. CBCT scans from a group of 50 patients were retrospectively screened, comprising 37 (74%) women and 13 (26%) men. The adult patients included in the study had a mean age of 42.4 (age between 30.3 and 55.8) and a mean treatment period of 23 months (minimum treatment time of 14 months and maximum treatment time of 40 months).

The inclusion criteria for this study were as follows:

- Healthy adults (with completed development phase);
- No history of trauma;
- No previous orthodontic treatment;
- No periodontal disease;
- No significant illness related to bone metabolism;
- No movement or metal artifact on the CBCT images;
- Good quality and contrast imaging;
- Presence of at least a central incisor (CI), a first premolar (1PM), and a first molar (1M) in each dental arch;
- Patient underwent fixed orthodontic treatment with or without upper first or second premolar extraction cases using Roth prescription brackets (American Orthodontics, Sheboygan, WI, USA).

The exclusion criteria were as follows:

- Incomplete or sectional CBCT imaging;
- Previous orthodontic treatment;
- Systemic disease or medications that would influence the outcome of treatment;
- Patients that could not keep up with regular appointments during treatment;
- Failure to follow oral hygiene protocols during treatment (OHI).

#### 2.1.1. Cone Beam Computed Tomography (CBCT)

The CBCT scans of the patients included in the study were captured using KaVo OP 3D equipment (Kavo Ltd., Charlotte, NC, USA), with the following settings: KaVo OP 3DI tube voltage of 90 kV, tube current of 6 mA, field of view (FOV) of 8 × 15 cm, and standard resolution (voxel size 200 µm). Each scan was performed with the subject in a seated upright position, with the Frankfurt plane parallel to the floor, while the midsagittal plane was perpendicular to the ground, in maximum intercuspation. The OnDemand 3D dental software, version 1.0 (Cybermed, Daejeon, Republic of Korea), with DICOM format, was used to view and analyze the parameters required for the study. CBCT imaging was recorded before (T0) and after (T1) orthodontic treatment. The measurements were performed in a blinded manner by one of the authors in order to avoid measurement bias. T1 measurements were performed at different time points, without access to T0 values.

#### 2.1.2. Assessment of Alveolar Bone Level (ABL)

From a methodological point of view, the height of the alveolar crest was measured from the CEJ to the marginal ABL. The buccal–lingual (B-L) and mesial–distal (M-D) surfaces were each joined at the CEJ level, such that a reference line could be drawn with the aid of panoramic reformatting, consisting of drawing an arch at the CEJ level in the points of interest (most posterior, molars, premolars, canines and midline) bilaterally and at both jaws and MPR (multi-planar reformatting) in the sagittal and coronal planes. The level of alveolar bone was measured from the CEJ to the nearest marginal alveolar bone crest at buccal (B), lingual (L), mesial (M), and distal (D) surfaces of the following maxillary and mandibular teeth: central incisors (CIs), first premolars (1st PMs), and first molars (1st Ms).

- Buccal and lingual ABL (B/L-ABL) measurements were performed in the sagittal plane for anterior teeth and in the coronal plane for posterior teeth.
- Mesial and distal ABL (M/D-ABL) measurements were performed in the coronal plane for anterior teeth and in the sagittal plane for posterior teeth.

**Figure 1:**
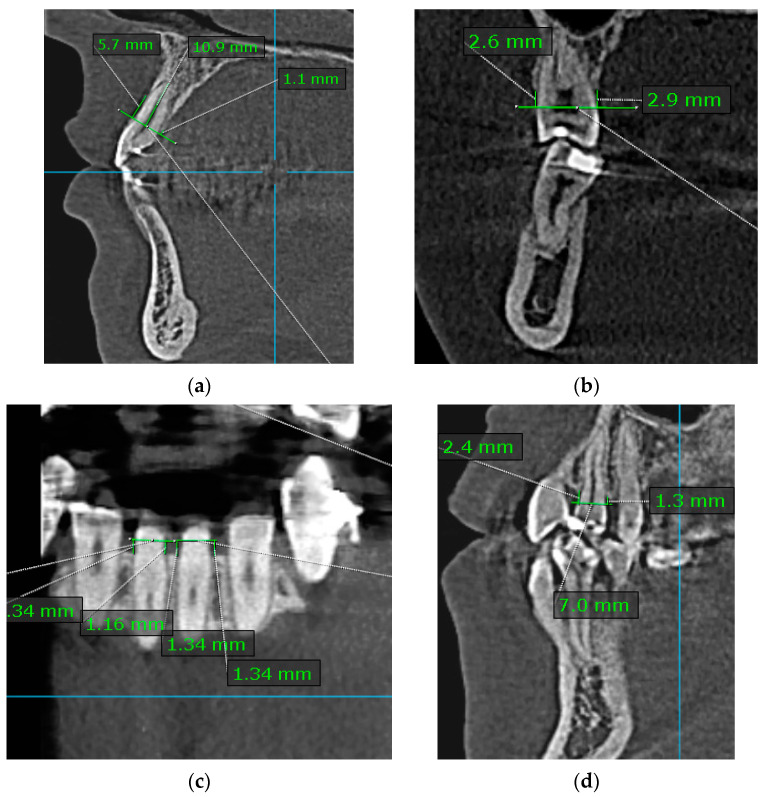
Example measurements for alveolar bone level (ABL). (**a**) B/L-ABL values measured in the sagittal plane for anterior teeth; (**b**) B/L-ABL values measured in the coronal plane for posterior teeth; (**c**) M/D-ABL values measured in the coronal plane for anterior teeth; (**d**) M/D-ABL values measured in the sagittal plane for posterior teeth. The respective lines were drawn perpendicular to the reference line placed at the CEJ.

Measurements were taken at a perpendicular distance to the reference line at the CEJ level, and the deepest point was evaluated when different levels of ABL at the same tooth were present (Figure 1a–d).

#### 2.1.3. Assessment of Root Length (RL)

The same reference line, drawn at the CEJ and joining at the buccal–lingual (B-L) surfaces used to assess ABL, was considered to evaluate the root length (RL). A vertical distance measurement was made along the long axis of the tooth/root, from the midpoint of the reference line at the CEJ to the most apical point of the root (CEJ-APEX).

**Figure 2:**
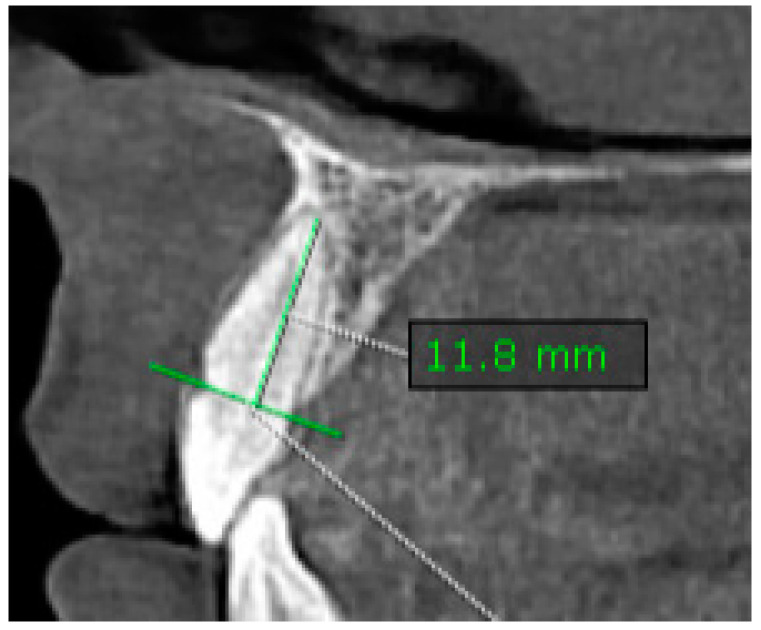
Example of measuring the RL (root length; CEJ-APEX) in the sagittal plane. A vertical distance measurement was made along the long axis of the tooth/root, from the midpoint of the reference line at the CEJ to the most apical point of the root (CEJ-APEX).

- Measurements were taken in the sagittal plane at the CIs of maxillary and mandibular teeth (Figure 2).

### 2.2. Data Collection

The difference in marginal ABL at the two times (T0, T1) was assessed in millimeters and evaluated at the buccal (B), lingual (L), mesial (M), and distal (D) surfaces of the teeth. Pre- and post-treatment (T0, T1) length measurements of the central incisors’ roots were also performed. The analysis included all the tooth surfaces obtained by CBCT for each patient. All measurements (B-ABL, L-ABL, M-ABL, D-ABL, CEJ-APEX) at each quadrant for the central incisor (CI), first premolar (1st PM), and first molar (1st M) were collected in a table, and supplementary data were also included (sex of the patient, duration of treatment, phase of treatment (T0, T1)).

### 2.3. Statistical Analysis

The statistical analysis in this study was performed using GraphPad Prism 8 software for macOS version 10.2.1 (GraphPad Software Inc., Boston, MA, USA). Median (Me) values were established for the right upper and lower (RU, RL) teeth, as well as the left upper and lower (LU, LR) teeth at T0 and T1. Mean values (M), standard deviations (SD), and standard errors of the mean were also assessed. The 95% confidence interval (CI) of the mean (lower and upper) was determined to examine the accuracy of measurement. The level of statistical significance was set at *p* < 0.05.

## 3. Results

Descriptive analysis was conducted for each measurement, following the example presented in Table 1.

The results revealed the presence of statistically insignificant differences in ABL between the values before and after treatment for the four tooth surfaces under consideration. Due to the non-normal distribution of the data, as confirmed through the Shapiro–Wilk test, and as the presence of heterogeneity of variances was confirmed for most of the results (paired) according to Levene’s test, the Wilcoxon test was selected for the initial analysis (Table 2 and Table 3).

**Figure 3:**
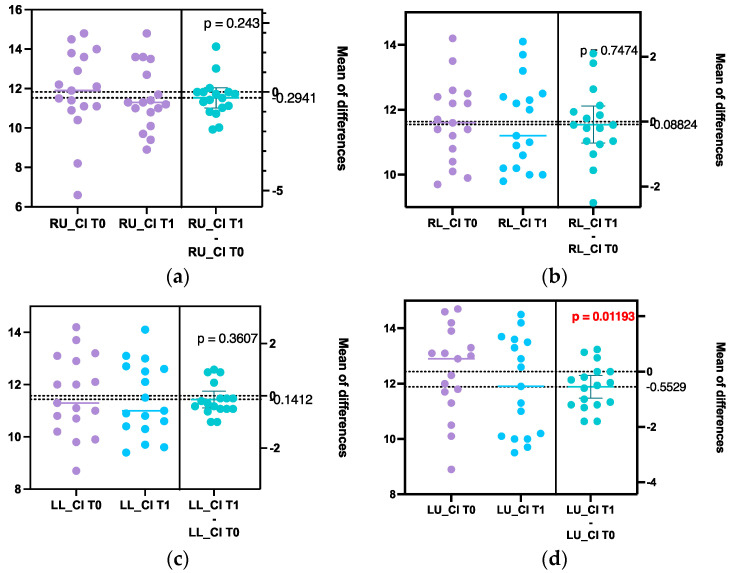
Distribution graph of the paired t-test for root resorption with *p*-value set at 0.05. Results at T0 and T1 for right upper central incisor (RU_CI) (**a**), right lower central incisor (RL_CI) (**b**), left lower central incisor (LL_CI) (**c**), and upper central incisor (LU_CI) (**d**).

For the measurements performed at the CEJ-APEX level, a paired t-test was applied due to the normal distribution of the data (Figure 3).

Overall, the post-treatment values for the measured parameters were decreased compared to the pre-treatment ones, although the changes were not statistically significant. However, at some levels (e.g., M-ABL, LL_CI, and RL_CI), an increase in the parameters was observed.

On the other hand, the outcome regarding root length (CEJ-APEX) was slightly different. The values showed no statistically significant differences at RU_CI, RL_CI, and LL_CI, while a noticeable decrease was observed at LU_CI (*p* = 0.01193), demonstrating increased root resorption after OT (Figure 3).

**Figure 4:**
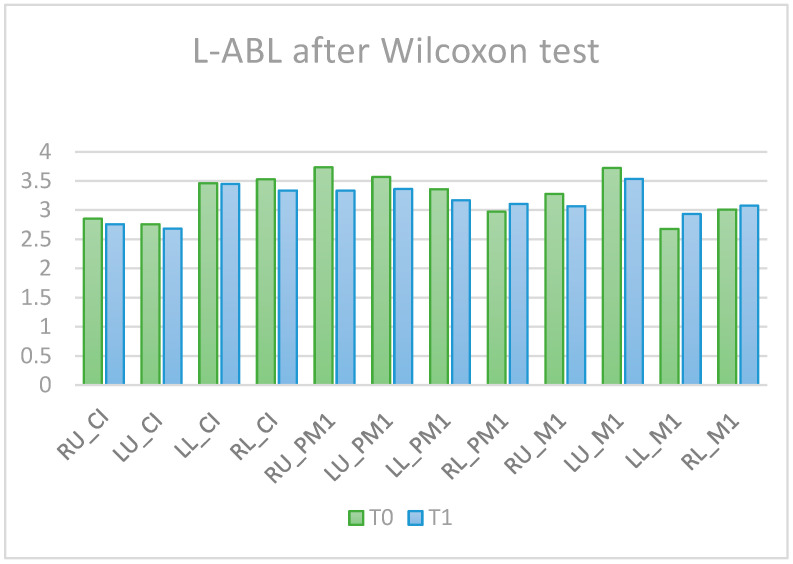
L-ABL before (T0) and after (T1) orthodontic treatment.

**Figure 5:**
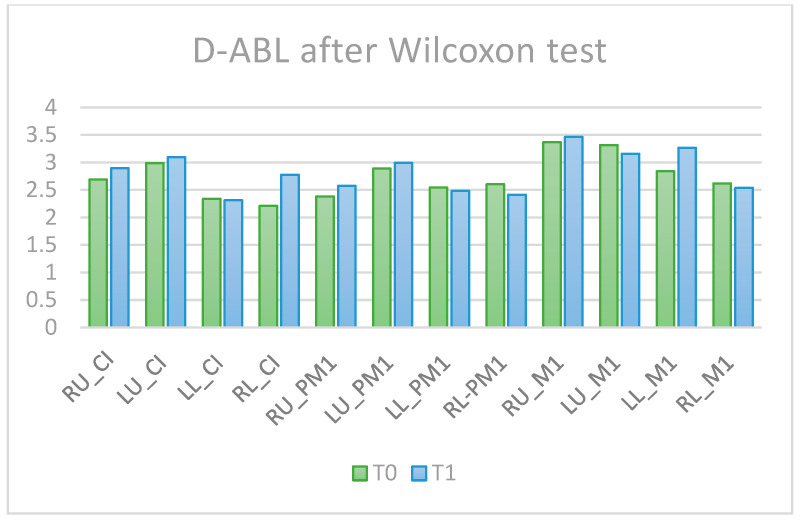
D-ABL before (T0) and after (T1) orthodontic treatment.

Comparisons of the alveolar bone levels at T0 and T1 are presented in Figure 4 for the L-ABL parameter and Figure 5 for the D-ABL parameter. In terms of L-ABL, the tooth RU_PM1 presented bone resorption and LL_M1 showed alveolar bone apposition after OT.

## 4. Discussion

This study aimed to analyze the changes in marginal alveolar bone height and root resorption pattern following fixed orthodontic treatment through 3D imaging. Due to the high-definition and detailed images provided through CBCT, accessibility to the four surfaces around the tooth was possible without the tendency to overlap, making it possible to obtain precise measurements of the vertical distance from the CEJ to marginal alveolar bone for evaluation, as well as measurements from the CEJ to the apex of the tooth [12,30,31,32,33,34].

The present study included healthy adult patients (74% female and 26% male) who followed fixed orthodontic treatment with or without extraction cases. The predominance of female patients could be justified by their increased interest in aesthetic perfection, making them more likely to start orthodontic treatment compared to the male population [13,36].

The results of our study did not show statistically significant differences at the level of the alveolar bone margin, even though more bone resorption was observed at the buccal surface (B-ABL) and lingual surface (L-ABL) of the right upper first premolar (RU_PM1), compared to the left lower first premolar (LL_PM1).

Furthermore, overall analysis of the B-ABL showed that the molars and incisors presented bone resorption, while the first premolar presented bone apposition. One possible explanation could be the amount of transversal movement of the molars, especially increased buccal crown torque, due to the Roth bracket prescription. Different torque values at the incisor level can influence the final positions of the teeth. In their study, Zhang et al. [18] observed different alveolar bone levels at the lower and upper central incisors. Lingual alveolar bone resorption at the mid-root was correlated with the movement of the mid-point of the lower incisor neck, while palatal alveolar bone resorption occurred at the apical level with the root apex point. Their explanation was that the movement types of the maxillary and mandibular central incisors are different in extraction cases where moderate anchorage is planned. Maxillary central incisors undergo bodily movement and their crowns tip due to the positive torque moment in the brackets, while mandibular central incisors present a tipping movement with little root movement. Unfortunately, one of the limitations of the present study is that the amounts of sagittal and transversal movements before and after the orthodontic treatment were not taken into consideration.

In contrast, on the distal surface (D-ABL) of the incisors, bone apposition was observed, where the right lower central incisor (RL_CI) presented the most marginal alveolar bone gain, while the right lower first premolar (RL_PM1) showed alveolar bone resorption. Similar results have been reported by Nahm et al. [16], who, in a retrospective study, assessed alveolar bone loss at the incisor level in Class I bidentoalveolar protrusion patients, also utilizing CBCT. Their results showed a higher percentage of alveolar bone loss in the lower incisors than in the upper incisors and, more specifically, on the lingual side for lower incisors and the buccal side for upper ones. Overall, the mean alveolar bone height loss happened to be consistent at lower anterior teeth compared to upper anterior teeth. Lower posterior teeth also showed a higher percentage of bone loss than upper posterior teeth. Following OT of bimaxillary protrusion cases, significant palatal bone loss can occur in the apical and mid-thirds of the root, as well as at the crestal level of the alveolar bone during the retrusion of the incisors and apex displacement with brackets [38].

In our study, an assessment of the alveolar bone levels was performed on patients with no signs of periodontal disease at T0 and T1. The study of Ma et al. focused on alveolar bone changes after fixed OT in patients with chronic periodontitis. Their results showed decreased alveolar bone height after OT when comparing the periodontally involved patients with healthy ones.

Another study by Jäger et al. [10] compared the periodontium reactions of two groups (young and adult) of patients after orthodontic treatment with fixed orthodontic appliances. The younger group presented reduced alveolar bone height on at least one tooth, while a lower bone height was found in both groups after treatment (especially in the mandible) overall. In our study, the age range of the patients was age between 30.3 and 55.8 years, including only adult patients. Changes after OT were also demonstrated in the study of Castro et al. [12], where the buccal surface of the lower CI was the most affected by bone loss (75%), while the lingual surface of the LI was least affected (40%). The buccal surface of the mandibular canines showed the greatest loss, leading to the conclusion that the morphology of the teeth and alveolar bone has a major influence on and acts as a limiting factor of orthodontic movement. In our study, the alveolar height at the level of the buccal surface of the CI was reduced; however, the changes were not statistically significant.

Zasčiurinskienė et al. [11] studied the outcome of periodontal–orthodontic treatment in terms of alveolar bone level changes in subjects with periodontal disease. No difference was observed between before and after OT, either in upper–lower incisors or anterior–posterior teeth comparisons; small changes in maxillary posterior teeth and a mean value of gain were seen, rather than loss. Loss was generally most commonly experienced on the buccal and lingual sides, rather than the mesial and distal sides, while gain was noted at the lingual side of maxillary retroinclined incisors.

Overall, in our study, major bone resorption appeared at the B and M surfaces of the teeth, while bone height increased after treatment (mainly at the D and L surfaces), followed by root resorption in the upper incisors.

The lack of statistical significance can be attributed to the heterogeneity of the data. This could be related to certain histological findings of bone resorption and apposition patterns during orthodontic treatment [39,40,41,42,43]. For this reason, the resorption process may precede, in compression areas, the formation of bone at tension areas [15]. Therefore, the orthodontic movement of a tooth in an area with decreased bone height can be achieved while preserving the height of the alveolar bone support consequent to the bone remodeling pattern [16,44].

Regarding the morphological changes of the alveolar bone after incisor tipping, some authors [45] have suggested that retraction of the maxillary incisor reduced the alveolar volume, both lingually and labially [45].

According to a meta-analysis conducted by Deng et al., root resorption measured in CBCT studies was more accurate and less significant than that established through two-dimensional research. The authors agreed that the evaluated evidence suggests that orthodontic treatment reduces the root length and volume. More evident root resorption has been observed on the maxillary lateral incisors, maxillary central incisors, and mandibular anterior teeth [22].

Major and careful attention should be given to the type and duration of movements generated by the forces acting on the teeth, the type of fixed appliance, and the class of malocclusion of the patients. Therefore, one of the limitations of the present study is that no differentiation between extraction and non-extraction cases was considered.

Thus, additional research is necessary to investigate the effects of bone adaptations and remodeling during OT, based on both skeletal characteristics and Angle’s classification of malocclusion, taking into consideration that variations in the jaw structure and mandibular plane angle alter the morphology and the alveolar process. In addition, it seems essential to evaluate the influence that different fixed orthodontic systems and mechanisms of treatment have on teeth and bone structures, including the effects of accelerated tooth movement techniques.

## 5. Conclusions

To conclude, on the basis of the evaluation of alveolar bone after orthodontic treatment in healthy patients presented in this study, it can be stated that orthodontic treatment maintains alveolar bone height, although it is more likely to cause root resorption, especially in the upper central incisors.

## Figures and Tables

**Table 1:** Example of descriptive statistics for B-ABL. The median, mean, standard deviation, and confidence interval data are included (RU: right upper; LU: left upper; LL: left lower; RL: right lower). The same analysis was also conducted for L-ABL, M-ABL, D-ABL, and CEJ-APEX.

Treatment Phase		Minimum	Maximum	Mean	Std. Deviation	Std. Error of Mean	Lower 95% CI of Mean	Upper 95% CI of Mean
Pre-treatment	RU_CI	1.3	5.7	2.853	1.039	0.2383	2.352	3.353
	LU_CI	1.3	4.5	2.758	0.8167	0.1874	2.364	3.152
	LL_CI	1.3	6.1	3.463	1.311	0.3008	2.831	4.095
	RL_CI	1.8	5.8	3.529	1.438	0.3389	2.814	4.244
	RU_PM1	1.8	6.5	3.735	1.234	0.2994	3.101	4.37
	LU_PM1	1.2	5.5	3.569	1.138	0.2846	2.962	4.175
	LL_PM1	1.8	5	3.358	0.9131	0.2095	2.918	3.798
	RL_PM1	1.5	6	2.974	1.171	0.2686	2.409	3.538
	RU_M1	2.6	5.4	3.275	0.7921	0.2287	2.772	3.778
	LU_M1	1.5	5	3.723	0.8918	0.2473	3.184	4.262
	LU_M1	2.2	3.1	2.68	0.3421	0.153	2.255	3.105
	RL_M1	1.8	5.3	3.01	0.9643	0.3049	2.32	3.7
Post-treatment	RU_CI	0.5	6.1	2.759	1.386	0.3361	2.046	3.471
	LU_CI	1.3	5	2.682	0.8719	0.2115	2.234	3.131
	LL_CI	1.6	6.6	3.447	1.285	0.3117	2.786	4.108
	RL_CI	1.6	7.1	3.335	1.516	0.3677	2.556	4.115
	RU_PM1	1.6	5.6	3.333	1.056	0.2727	2.749	3.918
	LU_PM1	1.5	5.9	3.362	1.226	0.34	2.621	4.102
	LL_PM1	1.7	6.2	3.167	1.213	0.2859	2.563	3.77
	RL_PM1	2	4.4	3.106	0.8111	0.2028	2.674	3.538
	RU_M1	2	4.9	3.067	0.9206	0.3069	2.359	3.774
	LU_M1	0.8	5.4	3.533	1.162	0.3354	2.795	4.271
	LL_M1	2	3.6	2.933	0.8327	0.4807	0.8649	5.002
	RL_M1	1.6	5	3.075	1.229	0.4346	2.047	4.103

**Table 2:** Wilcoxon test results for B-ABL.

		Mean			Mean	*p*-Value
Pre-treatment	RU_CI	2.853	Post-treatment	RU_CI	2.759	0.5398
	LU_CI	2.758		LU_CI	2.682	0.8499
	LL_CI	3.463		LL_CI	3.447	0.9438
	RL_CI	3.529		RL_CI	3.335	0.7259
	RU_PM1	3.735		RU_PM1	3.333	0.1177
	LU_PM1	3.569		LU_PM1	3.362	0.6719
	LL_PM1	3.358		LL_PM1	3.167	0.2729
	RL_PM1	2974		RL_PM1	3.106	0.2458
	RU_M1	3.275		RU_M1	3.067	0.6328
	LU_M1	3.723		LU_M1	3.533	0.3477
	LL_M1	2.68		LL_M1	2.933	0.5179
	RL-M1	3.01		RL_M1	3.075	0.9493

**Table 3:** Wilcoxon test results for M-ABL.

		Mean			Mean	*p*-Value
Pre-treatment	RU_CI	2.947	Post-treatment	RU_CI	2.665	0.4466
	LU_CI	3.821		LU_CI	2.412	0.1674
	LL_CI	2.405		LL_CI	2.683	0.5356
	RL_CI	2.383		RL_CI	2.676	0.2781
	RU_PM1	2.529		RU_PM1	2.627	0.9890
	LU_PM1	2.9		LU_PM1	2.815	0.9268
	LL_PM1	2.963		LL_PM1	2.574	0.1349
	RL-PM1	2.8		RL-PM1	2.794	0.4711
	RU_M1	3.092		RU_M1	3.018	>0.9999
	LU_M1	2.885		LU_M1	2.842	0.8411
	LL_M1	2.04		LL_M1	2.467	0.3929
	RL-M1	2.28		RL-M1	2.213	0.6250

## Data Availability

The data presented in this study are available on request from the corresponding author.
